# The Meaning of Diagnosis for Different Designations in Talking About Autism

**DOI:** 10.1007/s10803-020-04584-3

**Published:** 2020-06-30

**Authors:** Ralf Tepest

**Affiliations:** Psychiatry and Psychotherapy, University of Cologne, Faculty of Medicine and University Hospital Cologne, Kerpener Str. 62, 50937 Köln, Germany

This letter refers to a discussion initiated by a query to JADD. Therein the question was asked: How should we talk about individuals with a diagnosis of autism? Could we provide arguments for choosing one of the two formulations: “person with autism” or “autistic person”? In the response Vivanti (2020) described that the preferred designation changed over time as society’s view of people with disabilities has changed. Seen historically, the change in language reflects changes in society. Vivanti advocates to take this into account and additionally recommends to consult participants in each scientific study about their actual preferences. He concluded that JADD should support the judicious use of person-first and identity-first language in the papers as appropriate for the context.

I fully agree with the conclusion, that favors a differentiated terminology in talking about autism, but would like to highlight another aspect, which in my opinion should be a strong argument for differentiated use of terms apart from what is addressed in the afore cited statement.

My point is, that it is of central importance that the assignment of a certain diagnosis has a substantially different meaning for the affected individuals versus the clinical and scientific professionals facing an affected person.

For professionals in medicine, any diagnosis is characterized by a detectable consistency in specific symptoms and signs of different persons. The shared attributes that define a disease are relevant. In contrast any other characteristics of the individuals can be disregarded. Therefore common symptoms are in focus in any clinical intervention and not diverse other non-relevant individual properties.

This leads to a separate consideration of illness on the one hand and person as such on the other hand. Linguistically a clean separation can be achieved most easily by naming the affected person as a composite: person with autism, person with a virus infection, etc.

On the contrary, for an affected person a diagnosis has a completely different meaning: It enables any individual to explain aspects of his or her own identity. An example with close analogy to autism might be nearsightedness.

In a world of normal sighted people, a person with myopia whose condition is undiagnosed might experience that others are doing puzzling things. While all others are capable to react, for example, to displayed signs, the nearsighted person may not even recognize that anything was displayed. As a consequence the undiagnosed nearsightedness will evoke the impression of being different.

Here the diagnosis offers an explanation: the cause for the experienced otherness. In consequence of the diagnosis an appropriate intervention might follow.

While in nearsightedness suited glasses might help to enter another world, in conditions like total blindness there is no way to get the others’ view. People born blind can perceive their surroundings only from the perspective of a blind person. For them it is not possible to toggle to a person’s view without blindness. The specific condition of an affected person is inseparably linked to the experienced identity. Grammatically this can be implemented by adding a preceding attribute: blind person, autistic person, etc.

To sum up: To answer the question whether “person with autism” or “autistic person” is the adequate term, might depend on one’s own perspective. Therefore it is plausible that an approach that investigates how affected people, parents or professionals would like to talk about autism found diverging preferences (Kenny et al. 2016). Taken this together with the points made above, any divergence of the used terms is not a shortcoming, but shows that different perspectives should be adequately represented.
